# Editorial: Gender Bias in Publishing: Double-Blind Reviewing as a Solution?

**DOI:** 10.1523/ENEURO.0225-18.2018

**Published:** 2018-06-26

**Authors:** Christophe Bernard

Many studies, commentaries, blogs, etc. point at a gender bias in favor of males for award and acceptance of both grants and publications, respectively (e.g., Wenneras and Wold, 1997; Larivière et al., 2013 and Barres, 2006). According to biaswatchneuro's calculations for the registration at the 2017 Society for Neuroscience Annual Meeting, the female/male faculty ratio was 39%, and the ratio for trainees was 49%. This difference suggests the existence of gender bias in neuroscience. Of course, there is a time lag between being a trainee and becoming a faculty member, but parity has existed for decades at the trainee level (50% of PhDs in Neuroscience have been earned by women in the US since 1990). Yet, this does not translate at the faculty level. I believe that this issue needs to be solved at the faculty recruitment stage. As a society journal, there is not much that we can really do, except continue to alert the community to this issue.

However, scientific journals may take actions to help mitigate gender bias at the publication. If we consider the present 39% female/male ratio for faculty positions, unbiased representation means that there should be 39% females as corresponding/senior authors.

Biaswatchneuro released their latest numbers for January to March 2018 for the *Journal of Neuroscience*, *Neuron*, *Nature Neuroscience*, and *eNeuro*. All journals are below the anticipated 39%, although the two non-profit society journals, *Journal of Neuroscience* and *eNeuro*, are doing better than the other two. Recent studies demonstrate a persistent underrepresentation of female authors in scientific journals (Lariviere et al, 2013; Bendels et al, 2018; Pezzoni et al, 2016). Among neuroscience journals, the proportion of female authors is negatively correlated with impact factor (Shen et al, 2018). These numbers provide facts, although it is difficult to interpret them. Where is the bias coming from? Many interpretations have been proposed, such as instances when female PIs may be more cautious, only submitting papers when they feel the story is really complete, and bias coming from the journal. As a journal, we can act on the last parameter.

In most neuroscience journals, when papers are submitted, they are assigned to a reviewing editor, who then assigns them to reviewers. Is there a gender bias at that stage? The literature on gender bias shows that both men and women exhibit bias against female scientists (Raymond, 2013). This could be, behind the results posted by biaswatchneuro regarding neuroscience publications. But critical numbers are missing, those at submission. If a journal is successfully avoiding the effects of gender bias during the peer-review process, then the female/male ratio at publication must be the same as the ratio at submission. If not, it means that a bias has been introduced during the reviewing process.

We thus conducted a survey at *eNeuro* for all papers submitted/published since January 2015. The female/male ratio at submission is 30% … and the ratio at acceptance is 30%! The journal *eNeuro* is publishing neuroscience papers in which the gender ratio of the submitting authors matches the gender distribution of published authors. It will be interesting to share and collate numbers with the other Neuroscience journals.

Regarding first authors, the ratios are similar: 45% at submission and acceptance, close to the aforementioned 49% at the trainee level. I do not know why *eNeuro* does not receive 39% of submissions from female last authors, and I hope that this will change soon.

I can attempt to interpret why we have a balance between submission and acceptance that avoids gender bias. At *eNeuro*, we use a double-blind review system. That is to say, any information that explicitly identifies the authors is removed at the submission stage. Of course, the system cannot be perfect (e.g., authors have presented their unpublished results at conferences), and it is not supposed to be perfect. It removes unconscious bias whenever possible (a win-win situation). Hence, in most instances, the reviewers do not know who the authors are, so their decision cannot be unconsciously biased by gender. Reviewers are forced to assess the science itself, without relying upon author gender, reputation, institution, or country of origin, etc. The only scientist who knows the gender of the authors is the reviewing editor. The board of reviewing editors at *eNeuro* is composed of roughly equal numbers of female and male scientists, reflecting the composition of the neuroscience workforce at the trainee level and representing an aspiration for a scientific workforce able to draw upon the creativity and intellectual effort of all scientists, regardless of gender. It is important to remember that the final decision of the reviewing editor is based on consensus reached during a consultation session with the reviewers who remained blind to the authors’ identity throughout the whole process.

My personal interpretation is that the double-blind review process minimizes gender bias during the evaluation of the paper. It avoids adding an additional layer of bias to the dissemination of research findings. Consequently, most, if not all, journals should adopt it.

It is possible that the double-blind review system solves, at the evaluation level, another important bias, that of early career versus established investigators, another area of potential disparity that *eNeuro* is keen to address.

## References

[B8] Barres BA (2006) Does gender matter? Nature 442:133–136. 10.1038/442133a 16848004

[B1] Bendels MHK, Müller R, Brueggmann D, Groneberg DA (2018) Gender disparities in high-quality research revealed by Nature Index journals. PLoS One 13:e0189136 10.1371/journal.pone.0189136 29293499 PMC5749692

[B2] Larivière V, Ni C, Gingras Y, Cronin B, Sugimoto CR (2013) Global gender disparities in science. Nature 504:211–213. 10.1038/504211a 24350369

[B3] Meyer M, Cimpian A, Leslie S-J (2015) Women are underrepresented in fields where success is believed to require brilliance. Front Psychol 6:235 10.3389/fpsyg.2015.00235 25814964 PMC4356003

[B4] Pezzoni M, Mairesse J, Stephan P, Lane J (2016) Gender and the publication output of graduate students: A case study. PLoS One 11:e0145146 10.1371/journal.pone.0145146 26760776 PMC4711938

[B5] Raymond J (2013) Most of us are biased. Science (80) 495:33–34. 10.1038/495033a 23467152

[B6] Shen YA, Webster JM, Shoda Y, Fine I (2018) Persistent Underrepresentation of Women's Science in High Profile Journals. bioRxiv 275362 10.1101/275362

[B7] Wenneras C, Wold A (1997) Nepotism and sexism in peer-review. Nature 10.1038/387341a0 9163412

